# Peri-treatment change of anorectal function in patients with rectal cancer after preoperative chemoradiotherapy

**DOI:** 10.18632/oncotarget.20567

**Published:** 2017-08-27

**Authors:** Jin Sook Song, In Ja Park, Jeong Hye Kim, Hyang Ran Lee, Jeong Rang Kim, Jong Lyul Lee, Yong Sik Yoon, Chan Wook Kim, Seok Byung Lim, Chang Sik Yu, Jin Cheon Kim

**Affiliations:** ^1^ Department of Colorectal Clinic, Asan Medical Center, Seoul, Korea; ^2^ Department of Colon and Rectal Surgery, Asan Medical Center, University of Ulsan College of Medicine, Seoul, Korea; ^3^ Department of Clinical Nursing, University of Ulsan, Seoul, Korea

**Keywords:** anorectal manometry, anorectal function, preoperative chemoradiotherapy, rectal cancer

## Abstract

Preoperative chemoradiotherapy (PCRT) is a standard treatment for locally advanced rectal cancer. The influence of PCRT on anorectal function has not been objectively assessed. We evaluated the short-term influence of PCRT on anorectal function in patients with locally advanced rectal cancer using anorectal manometry. We included 310 patients with locally advanced mid and lower rectal cancer who underwent PCRT from 2012 to 2015. We compared anorectal function based on anorectal manometry between before and after PCRT according to tumor location, clinical T (cT) stage, and tumor response after PCRT. Lower rectal cancer was common in the cohort of 310 patients (*n* = 228, 73.5%). Sphincter length (*p* = 0.003) and maximal resting pressure (*p* < 0.001) increased and maximal tolerated volume (*p* = 0.036) decreased after PCRT regardless of tumor location. Maximal squeezing pressure and rectal compliance slightly decreased, without statistical significance. Changes in manometric parameters after PCRT were not associated with changes of cT stage after PCRT. However, minimal sensory volume (*p* = 0.042) and maximal tolerated volume (*p* = 0.025) increased significantly in 143 patients (46.1%) with changes in the distance of the cancer from the anal verge after PCRT. PCRT did not impair the overall short-term anorectal manometric parameters in patients with locally advanced rectal cancer. Further study is required to investigate postoperative anorectal function after sphincter-preserving surgery to evaluate the long-term effects of PCRT on anorectal function.

## INTRODUCTION

Preoperative chemoradiotherapy (PCRT) is currently recommended as the standard treatment for locally advanced rectal cancer [1-3]. It is known to decrease local recurrence rates without increasing treatment-associated toxicity compared to postoperative adjuvant chemoradiotherapy [4-7]. In addition, PCRT has been reported to improve sphincter preservation in several studies [2, 5-7], and the introduction of PCRT has led to the greater possibility of sphincter preservation, especially in lower rectal cancer patients.

Many patients with rectal cancer wish to undergo sphincter-preserving surgery (SPS) to preserve quality of life. However, following SPS, defecation disorders such as frequent defecation, urgency, clustering, and fecal incontinence can occur, and cooperation of the rectum and anus is impaired. Therefore, anorectal function is compromised and quality of life is affected [8-11]. It is known that the possibility of SPS increases after PCRT, but there are conflicting opinions over whether SPS after PCRT causes more severe anorectal dysfunction than SPS. Previous studies have reported that patients with SPS after PCRT can develop fecal incontinence [3] due to anorectal functional deterioration [1, 3, 12-14] more frequently than patients who underwent surgery alone, and that PCRT causes damage to the anal sphincter and pudendal nerve [15].

Previous studies on the influence of PCRT on anorectal function have evaluated long-term functionality after SPS [1, 3, 12-14, 16, 17]. However, it is not easy to investigate the direct effects of PCRT on anorectal function since deterioration of anorectal function occurs when PCRT is combined with SFS [8, 18, 19].

With the above in mind, the objective of the present study was to exclude the effect of SPS in assessing the influence of PCRT on anorectal function by comparing changes after PCRT using anorectal manometry.

## RESULTS

### Characteristics of patients

The mean age of the 310 patients was 60.2 ± 11.2 years (range, 29-83 years). The male/female ratio was 2:1. The mean distance of the tumor from the anal verge (AV) was 4.48 ± 1.36 cm, and lower rectal cancer patients were predominant in the cohort. Upon clinical staging, 281 patients (90.6%) were T3 and 229 patients (73.9%) were N2. Tumors with moderate differentiation upon biopsy were the most common. Three patients had undergone temporary ileostomy before PCRT. The median dose of radiation was 50.4 Gy (50-50.4 Gy). The most frequently used concurrent chemotherapy agents were 5-fluorouracil (FU) and leucovorin (LV) (Table 1).

**Table 1:** General and clinical characteristics of patients

Variables	Values, *n* (%) or mean ± SD
Age, years (range)	60.2 ± 11.2 (29–83)
Sex	
Male	207 (66.8)
Female	103 (33.2)
Location of tumor, cm from AV	4.48 ± 1.36
LR (≤ 5 cm)	228 (73.5)
MR (> 5 cm, ≤ 8 cm)	82 (26.5)
Clinical T stage	
T3	281 (90.6)
T4	29 (9.4)
Clinical N stage	
N0	10 (3.2)
N+	300 (96.8)
Histologic differentiation	
Well differentiated	79 (25.5)
Moderately differentiated	224 (72.2)
Poorly differentiated	7 (2.3)
Clinical symptom	
Hematochezia or Anal bleeding*	251 (81.0)
Anal pain*	45 (14.5)
Tenesmus*	119 (38.4)
Stool caliber change*	206 (66.5)
Number of defecations/day	3.28 ± 2.88
Temporary Ileostomy	3 (1.0)
Radiation dose, GY, mean (range)	50.4 (50–50.4)
Concurrent chemotherapy	
5-FU + Leucovorin	208 (67.1)
Capecitabine	89 (28.7)
Capecitabine + Oxaliplatin	13 (4.2)

AV: anal verge, LR: lower rectum, MR: mid rectum, 5-FU: 5-fluorouracil

*multiple answers were included

Seventy-four patients (23.9%) experienced one or more treatment-associated adverse effect, but there were no effects over grade 3 according to the Common Toxicity Criteria. Among the adverse effects, anal pain was the most common (35 patients [11.3%]), followed by diarrhea in 11 patients (3.5%), radiation dermatitis in 10 patients (3.2%), nausea and vomiting in 10 patients (3.2%), dysuria in nine patients (2.9%), hand foot syndrome in six patients (1.9%), radiation proctitis in six patients (1.9%), constipation in three patients (1.0%), stomatitis in two patients (0.6%), and neurotoxicity in two patients (0.6%).

### Changes in clinical characteristics between before and after PCRT

After PCRT, the distance of the tumor from the AV increased significantly compared to that before PCRT. Among the 310 patients, the mean distance increased by 0.94 ± 0.09 cm after PCRT in 143 patients (46.1%), but it was unchanged in 109 patients (35.2%) and decreased in 58 patients (18.7%). Down-staging by magnetic resonance imaging (MRI) was observed in 100 patients (32.3%). After PCRT, 32 patients (10.3%) were down-staged in clinical T (cT) stage, 88 patients (28.4%) were down-staged in clinical N (cN) stage, and 20 patients (6.5%) were down-staged in both cT and cN stage.

Among clinical symptoms, hematochezia or anal bleeding, tenesmus, and stool caliber decreased significantly at 4 weeks after the completion of PCRT, while anal pain increased significantly. The average number of defecations per day in the 307 patients (excluding three patients [1.0%] who had undergone temporary ileostomy) decreased significantly after PCRT (Table 2).

**Table 2:** Clinical characteristics between before and after PCRT

Variables	Before PCRT	After PCRT	*p*-value
Location of tumor, cm from AV	4.48 ± 1.36	4.76 ± 1.46	<.001
LR (≤ 5 cm)	228 (73.5)	211 (68.1)	.016
MR (> 5 cm, ≤ 8 cm)	82 (26.5)	99 (31.9)	
Clinical T stage			
T2	0 (0.0)	26 (8.4)	<.001
T3	281 (90.6)	254 (81.9)	
T4	29 (9.4)	30 (9.7)	
Clinical N stage			
N0	10 (3.2)	38 (12.3)	<.001
N+	300 (96.8)	272 (87.7)	
Clinical symptom			
Hematochezia or Anal bleeding*	251 (81.0)	7 (5.5)	<.001
Anal pain*	45 (14.5)	92 (29.7)	<.001
Tenesmus*	119 (38.4)	37 (11.9)	<.001
Stool caliber change*	206 (66.5)	11 (3.5)	<.001
Number of defecations/day	3.28 ± 2.88	2.44 ± 2.35	<.001

PCRT: preoperative chemoradiotherapy, AV: anal verge, LR: lower rectum, MR: mid rectum

*multiple answers were included

### Changes in manometric parameters between before and after PCRT

There were significant differences in sphincter length (SL), maximal resting pressure (MRP), and maximal tolerated volume (MTV) among the manometric parameters after PCRT. Maximal squeezing pressure (MSP) and rectal compliance slightly decreased without statistical significance (Figure 2).

**Figure 1:**
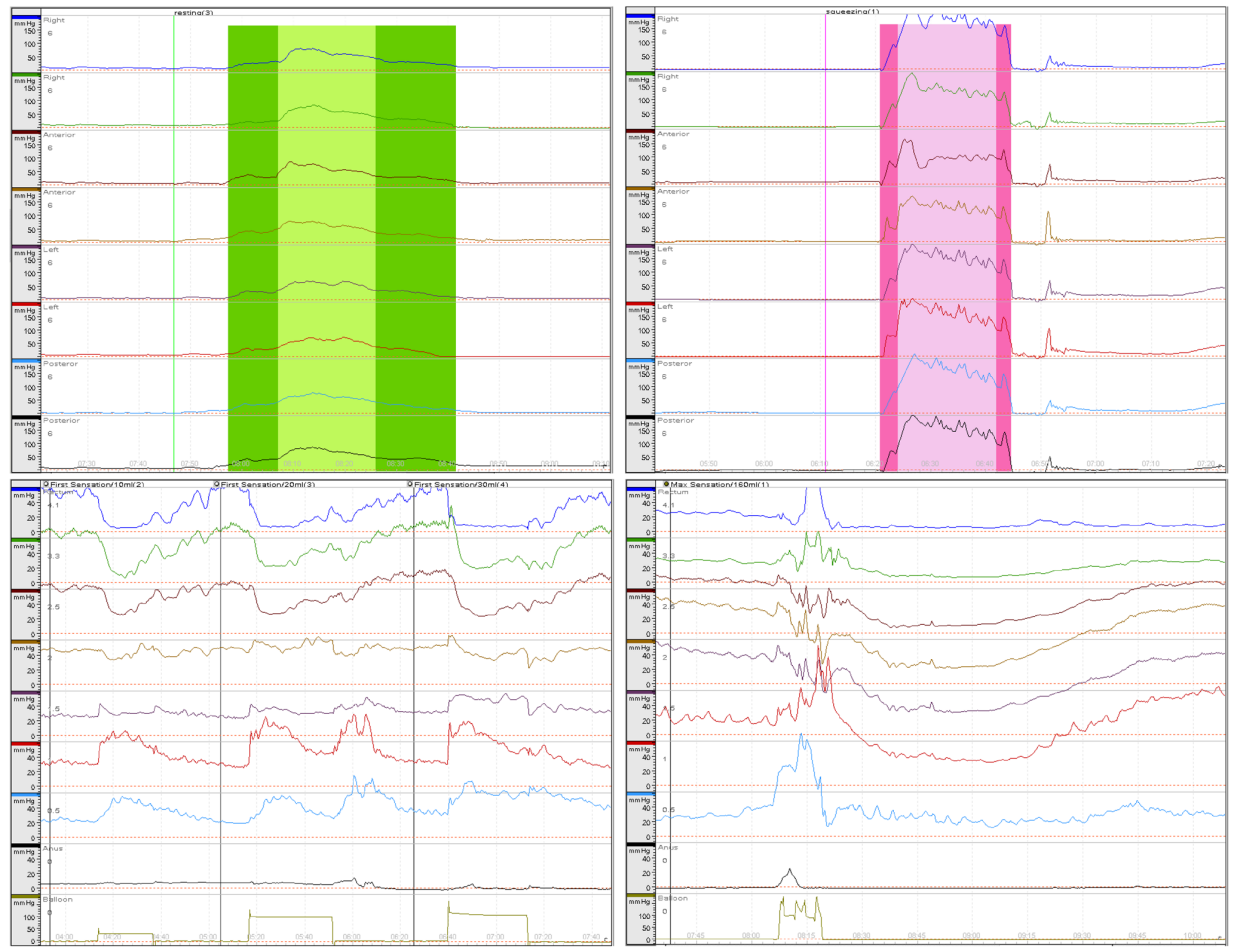
Anorectal manometry evaluation graph Sphincter length, high pressure zone length, anal sphincter symmetry index, and maximal resting pressure (left top); maximal squeezing pressure (right top); minimal sensory volume and rectoanal inhibitory reflex (left bottom); urgent volume and maximal tolerated volume (right bottom). All measured using a hydraulic capillary infusion system.

**Figure 2:**
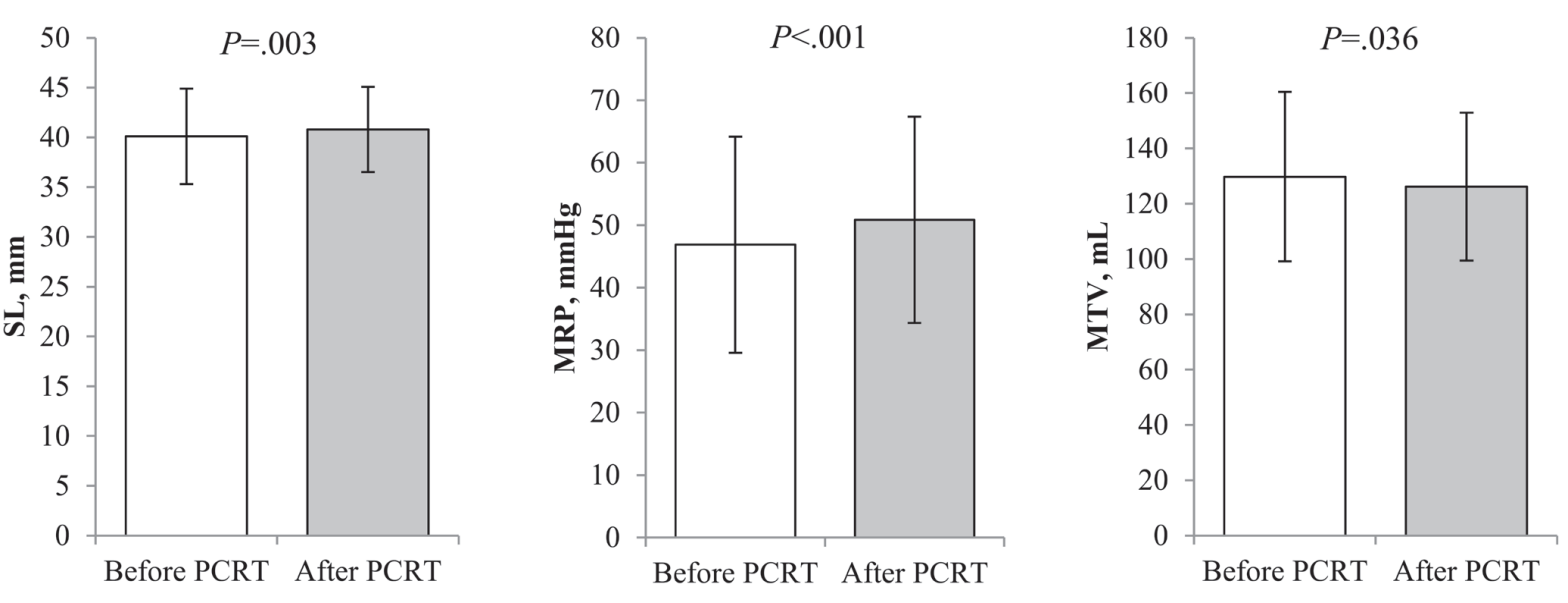
Comparison of manometric parameters between before and after preoperative chemoradiotherapy Sphincter length (40.1 ± 4.8 *vs*. 40.8 ± 4.3 mm, *p* = 0.003) and maximal resting pressure (46.88 ± 17.29 vs. 50.88 ± 16.50 mmHg, *p* < 0.001) were significantly increased after PCRT. Maximal tolerated volume decreased after PCRT (129.77 ± 30.65 vs. 126.19 ± 26.71 mL, *p* = 0.036).

When comparing the differences in the location of the tumors, the SL and MRP increased significantly in both lower and mid rectal cancers. While the MTV and rectal compliance slightly decreased, these differences were not statistically significant. Changes between manometric parameters before and after PCRT were not associated with the location of tumor. When comparing changes in the clinical stages of the tumors, SL and MRP showed significant increases while MTV decreased significantly after PCRT in cT3 rectal cancers. However, there were no significant changes in cT4 rectal cancers between before and after PCRT (Table 3).

**Table 3:** Comparison of manometric parameters between before and after PCRT according to tumor characteristics

Variables	Location of tumor						Clinical T stage					
	LR(≤5 cm) (*n* = 228)			MR(>5 cm) (*n* = 82)			cT3 (*n* = 281)			cT4 (*n* = 29)		
	Before PCRT	After PCRT	*P*	Before PCRT	After PCRT	*P*	Before PCRT	After PCRT	*P*	Before PCRT	After PCRT	*P*
SL ^*^	4.00±0.49	4.06±0.44	.020	4.05±0.43	4.13±0.42	.044	3.98±0.46	4.08±0.43	<.001	4.26±0.56	4.13±0.46	.217
HPZ ^*^	2.22±0.62	2.26±0.58	.391	2.24±0.57	2.19±0.61	.554	2.22±0.60	2.25±0.59	.385	2.30±0.68	2.13±0.56	.285
MRP ^+^	46.86±17.64	50.83±16.23	<.001	46.95±16.38	51.02±17.33	.012	45.70±16.67	50.43±16.39	<.001	58.33±19.23	55.29±17.23	.325
MSP ^+^	178.64±94.43	170.59±70.68	.126	175.36±70.95	178.81±67.51	.523	174.67±89.80	170.84±68.37	.380	207.88±72.03	191.33±81.80	.198
ASI	0.80±0.08	0.81±0.66	.652	0.80±0.07	0.81±0 .07	.132	0.80±0.08	0.80±0.07	.489	0.81±0.07	0.84±0.07	.129
MSV^§^	10.79±3.01	10.57±2.32	.385	10.49±2.17	10.00±0.00	.045	10.71±2.84	10.39±1.94	.128	10.69±2.58	10.69±2.58	1.00
UV ^§^	59.34±9.00	59.21±6.37	.841	59.88±9.88	59.82±6.78	.962	59.79±9.29	59.48±6.63	.630	56.55±8.14	58.28±4.68	.283
MTV^§^	127.61±30.91	125.42±26.06	.233	135.79±29.28	128.35±28.48	.062	130.55±30.70	126.90±26.62	.048	122.24±29.69	119.31±27.05	.468
RC^**£**^	1.65±1.40	1.47±1.23	.137	1.64±1.03	1.49±0.91	.288	1.63±1.13	1.47±1.18	.077	1.79±2.46	1.56±0.86	.640
RAIR¶	92.1	94.7	.286	92.7	96.3	.375	91.5	95.0	.076	100	96.6	

^*^cm, ^+^ mmHg, ^§^mL; ^￡^ - mL/mmHg, ¶%

PCRT: preoperative chemoradiotherapy, LR: lower rectum, MR: mid rectum, SL: sphincter length, HPZ: high pressure zone, MRP: maximal resting pressure, MSP: maximal squeezing pressure, ASI: anal sphincter symmetry index, MSV: minimal sensory volume, MTV: maximal tolerated volume, UV: urgent volume, RC: rectal compliance, RAIR: rectoanal inhibitory reflex

We investigated changes in manometric parameters according to the changes in distance from the AV and cT stage after PCRT. Minimal sensory volume (MSV) and MTV increased significantly in 143 patients (46.1%) who had greater distance of the tumor from the AV after PCRT. However, in patients with no increase in distance or a smaller distance of the tumor from the AV after PCRT, MSV and MTV showed significant decreases after PCRT. There were no significant differences in manometric parameters after PCRT according to down-staging in cT stage after PCRT (Table 4).

**Table 4:** Comparison of the differences in manometric parameters between before and after PCRT according to tumor response to PCRT

Variables	Tumor distance from the anal verge			Clinical T stage		
	No increase (*n* = 167)	Increase (*n* = 143)	*p*-value	Not reduced (*n* = 278)	Reduced (*n* = 32)	*p*-value
SL, cm	0.07 ± 0.46	0.08 ± 0.40	.795	0.07 ± 0.44	0.08 ± 0.39	.967
HPZ length, cm	-0.01 ± 0.71	0.05 ± 0.68	.511	0.04 ± 0.69	-0.17 ± 0.74	.127
MRP, mmHg	4.03 ± 14.26	3.95 ± 13.19	.959	4.04 ± 13.82	3.55 ± 13.41	.853
MSP, mmHg	-5.16 ± 89.66	-4.85 ± 45.06	.970	-6.59 ± 74.49	9.63 ± 48.41	.245
ASI	0.01 ± 0.09	0.01 ± 0.08	.983	0.00 ± 0.08	0.04 ± 0.13	.118
MSV, mL	-0.66 ± 3.66	0.14 ± 3.14	.042	-0.11 ± 2.99	-2.00 ± 6.10	.104
Urgent volume, mL	-0.69 ± 10.70	0.56 ± 9.91	.290	-0.02 ± 10.81	-1.00 ± 4.23	.622
MTV, mL	-7.10 ± 30.00	0.52 ± 29.56	.025	-4.07 ± 30.47	1.00 ± 25.00	.380
Rectal compliance, mL/mmHg	-0.29 ± 2.02	-0.03 ± 1.07	.174	-0.16 ± 1.50	-0.26 ± 2.83	.869

SL: sphincter length, HPZ: high pressure zone, MRP: maximal resting pressure, MSP: maximal squeezing pressure, ASI: anal sphincter symmetry index, MSV: minimal sensory volume, MTV: maximal tolerated volume

## DISCUSSION

The present study compared the results from before and after PCRT with the objective of assessing the direct influence of PCRT on anorectal function, for which changes in manometric parameters were observed as comparisons of objective indices. In addition, subgroup analysis was conducted to examine whether anorectal function is affected by changes in the clinical features of tumors after PCRT. According to the findings of the present study, anorectal manometry after PCRT revealed significant increases in SL and MRP and decreases in MTV.

There are varying reports regarding the changes in manometric parameters between before and after PCRT. In some studies [19, 20], SL increased as in the present study. In terms of pressure profiles, there have been contradictory reports [3, 20-22]. De Nardi et al. [22] reported that 23% of patients, showed the new onset of anorectal dysfunctions, mostly represented by a lower MRP after PCRT. They explained that it caused by radiation damage to the internal anal sphincter muscles. On the contrary, another study [20] reported increase in MRP after PCRT and we also showed increase in MRP after PCRT . There have been various hypotheses about radiation effect on defecatory function such as damage to anal sphincter injury, pudendal nerve, and reduced distensibility of the neo-rectum [15]. However, there was no clear explanation. Based on the results of the present study, radiation effect would not be detrimental to internal anal sphincter. Radiation effect on defecatory function was not mainly caused by sphincter damage but change in distensibility according to our results.

In the present study, a significant decrease in MTV was found subsequent to PCRT. Some studies [3, 19, 20] reported decreases in MTV after PCRT, but the differences were not statistically significant. Although some have also reported a decrease in rectal compliance [20], the present study did not find such a difference. Edema [20, 21] and acute inflammation [19, 21] in the anal and rectal mucosa caused by PCRT may cause pain in the anus and rectum, which may lead to increased tension in the internal anal sphincter and anorectal sensitivity, leading to changes in pressure or volume. However, such changes are not reported consistently. Such differences may arise from differences between patient groups, as well as the assessment methods and periods used in each study. However, the change in distensibility of neo-rectum may cause anorectal dysfunction after PCRT.

There have been attempts to compare the differences in anorectal function between before and after PCRT based on the location of the tumor. While one study [20] reported differences based on location when rectal cancer was differentiated as either lower and mid rectal cancer, other studies reported no differences in anorectal function [19, 21]. The present study also found no significant differences based on the location of the tumor, which is believed to be the result of both lower and mid rectal cancers being included in the radiation field. Furthermore, when any deterioration due to surgery was excluded, outcomes after PCRT did not vary based on the location of the tumor.

With respect to anorectal function after PCRT based on cT stages, significant increases in SL (*p* < 0.001) and MRP (*p* < 0.001) and significant decreases in MTV (*p* = 0.048) were found after PCRT in cT3 rectal cancer cases. However, there were no significant differences in anorectal function after PCRT in cT4 rectal cancer cases. It is believed that such differences may be attributable to the difference in sample size between cT3 (*n* = 281, 90.6%) and cT4 (*n* = 29, 9.4%) cases. However, some studies reported that tumor response to PCRT becomes worse in high cT stages [23-25]. Therefore, the possibility of changes in anorectal function based on cT stage being caused by such differences in responsiveness can also be suspected. In fact, among the participants in the present study, total regression was found in 45.2% of cT3 patients, which was higher than the 31.0% noted in cT4 patients. However, because of the difference in sample size between cT3 and cT4 rectal cancer cases, additional studies are needed to generalize the findings.

The changes in the distance of the tumor from the AV and cT stages after PCRT can be used as indirect indicators of tumor response to PCRT. The present study examined whether differences in tumor response to PCRT can cause changes in anorectal function based on indirect indicators. In patients who showed increased distance from the AV to the tumor after PCRT, there were significant increases in MSV and MTV, whereas in patients who showed no change or a decrease in the distance from the AV to the tumor, MSV and MTV decreased after PCRT. The former may be attributed to a decrease in tumor size. In the present study, there was no significant difference in anorectal function after PCRT based on changes in cT stage. Contrary to the findings in the present study, Kye *et al.* [19] showed significant increases in the length of the high pressure zone (HPZ) and urgent volume in down-staging of cT stage after PCRT. They claimed that such results were not attributable to improved rectal sensation, such as urgent volume, after PCRT in rectal cancer patients with down-staging cT stage, but rather due to exacerbation after PCRT in rectal cancer cases that did not show down-staging of cT stage. Due to the lack of accuracy in the evaluation of cT staging, it is believed that differences between studies may arise from difficulties in assessing actual responses to radiation by changes in cT stages.

In this study, clinical symptoms at 4 weeks after the completion of PCRT were mostly improved, but anal pain alone was significantly increased. Since anal pain may be caused by anal or rectal mucosal edema or ulceration from vasculitis near the tumor, as well as improved symptoms from a reduction in tumor size, these are also factors that may affect anorectal manometry and should be assessed as factors that affect anorectal function after radiation therapy. Based on the findings of the present study, MTV decreased after PCRT, but that decrease amounted to only 2.8% as compared to before PCRT, meaning that the difference was minimal. Meanwhile, there were no significant differences in rectal compliance and RAIR after PCRT, meaning that those factors may not have any short-term effects on exacerbating anorectal function. We only evaluated the anorectal dysfunction after PCRT using anorectal manometric finding. However, operative complications after PCRT such as anastomotic leakage may be associated with functional outcomes. In the present study, only 1% of patients received temporary diversion and leakage rate was less than 3%, therefore, the effect of complications and diversion on defecatory function would not be statistically evaluated. But, we need to consider these practical factors assessing the functional outcomes after PCRT.

Anorectal function after PCRT was similar regardless of the distance of the tumor from the AV, but there is a limitation in the interpretation of anorectal function according to cT stage due to differences in sample size. The anorectal function, however, have to be evaluated after definitive surgery because it would also influence on anorectal function. Indeed, the long-term effect of PCRT also has to be evaluated because anorectal function might be changed after definitive surgery and long term follow-up. This study included a relatively large sample of 310 patients with rectal cancer. However, this was a retrospective study. As such, it was limited because we were not able to use systematic assessment tools to measure fecal incontinence, constipation, and quality of life after PCRT. Further study is required to investigate postoperative anorectal function after SPS to evaluate the long-term influence of PCRT on anorectal function.

## MATERIALS AND METHODS

### Patients and preoperative chemoradiotherapy

We retrospectively reviewed 310 patients with locally advanced rectal cancer who underwent PCRT from January 2012 to May 2015 at Asan Medical Center. The participants were patients who had undergone anorectal manometry before and after PCRT and met the follow inclusion criteria: definitive diagnosis of adenocarcinoma from a biopsy, the tumor was located within 8 cm from the AV, and the tumor invasion level was T3-T4 or lymph-node metastasis was suspected on MRI. Tumor distance from the AV was measured using the curvilinear measurement on MRI by drawing multiple linear lines along the approximate luminal center of the rectum and the anus on the midline sagittal plane as recommended by a national recommendation. We excluded patients who were diagnosed with distant metastasis, had a history of radiotherapy or chemotherapy, could not complete PCRT, and were not available for anorectal manometry before and after PCRT. Rectal cancer was classified as either lower or mid rectal depending on the tumor being located ≤ 5 cm or > 5 cm to ≤ 8 cm from the AV, respectively. Patients received radiotherapy with 1.8-2.0 Gy per fraction at a total dose of 50-50.4 Gy in 25∼28 fractions using a 3 or 4-field technique. Concurrent chemotherapy was provided with an intravenous bolus of 5-FU and LV for 3 d after the beginning of radiotherapy and for 3 d before the end of radiotherapy, or with oral capecitabine twice daily during the treatment period.

This study was approved by the Institutional Review Board of the Asan Medical Center (authorization number 2016-0245).

### Anorectal manometry

Anorectal manometry was performed before PCRT and 4-8 weeks (mean, 5.96 ± 0.95 weeks) after the completion of PCRT. A hydraulic capillary infusion system (Polygraf ID, Medtronic, Denmark) was used with the pull-through technique, and the perfusion catheter was an 8-channel polyethylene flexible catheter with an outer diameter of 5.5 mm and a total length of 150 cm. A radial perfusion catheter was inserted so that the channel of the catheter would be positioned 6 cm from the AV. Subsequently, the SL, HPZ, MRP, and anal sphincter symmetry index (ASI) were measured while retracting with an automatic puller for 1 min at a constant rate of 1 mm/sec. After three repeated measurements under stable conditions, the catheter was reinserted and MSP was measured while squeezing the anal sphincter as much as possible in the HPZ while the catheter was being retracted continuously. A spiral perfusion catheter with a latex balloon attached at its end was inserted so that the #8 channel would be positioned on the AV. Subsequently, MSV, urgent volume, and MTV were measured as the latex balloon was inflated with a syringe, while at the same time checking for RAIR. The system-embedded software program (Polygram, USA) calculated SL, length of HPZ, MRP, MSP, ASI, and rectal pressure based on the measured pressure values, while rectal compliance was calculated based on the volume of the inflated air and rectal pressure (Figure 1). Anorectal manometry was performed by two nurses with more than 10,000 cases of experience with this procedure.

### Statistical analysis

We compared manometric parameters between before and after PCRT according to tumor characteristics, such as the location of the tumor, cT stage, and tumor response after PCRT. We measured the differences in manometric parameters between before and after PCRT, expressing increases as positive values and decreases as negative values. Quantitative manometric parameters except RAIR were expressed as the mean ± SD. Clinical characteristics except the number of defecations between before and after PCRT were compared using the chi-square test (McNemar test). The paired t-test and chi-square test (McNemar test) were used to compare manometric parameters between before and after PCRT. Statistical analyses were performed using SPSS version 22.0 (IBM Statistics, Armonk, NY). *p*-values < 0.05 were considered statistically significant.

## Abbreviations

PCRT: preoperative chemoradiotherapy
SPS: sphincter-preserving surgery
AV: anal verge
SL: sphincter length
HPZ: high pressure zone
MRP: maximal resting pressure
ASI: and anal sphincter symmetry index ASI
MTV: maximal tolerated volume
MSV: minimal sensory volume
MSP: maximal squeezing pressure
RAIR: rectoanal inhibitory reflex
FU: fluorouracil
LV: leucovorin
